# Supplementary material for: Optical manipulation of single flux quanta
Source: Nat Commun. 2016 Sep 28;7:12801. doi: 10.1038/ncomms12801 (PMC5052701; doi:10.1038/ncomms12801)
Supplement: Supplementary Information — Supplementary Figures 1-4, Supplementary Notes 1-5 and Supplementary References [file ncomms12801-s1.pdf]

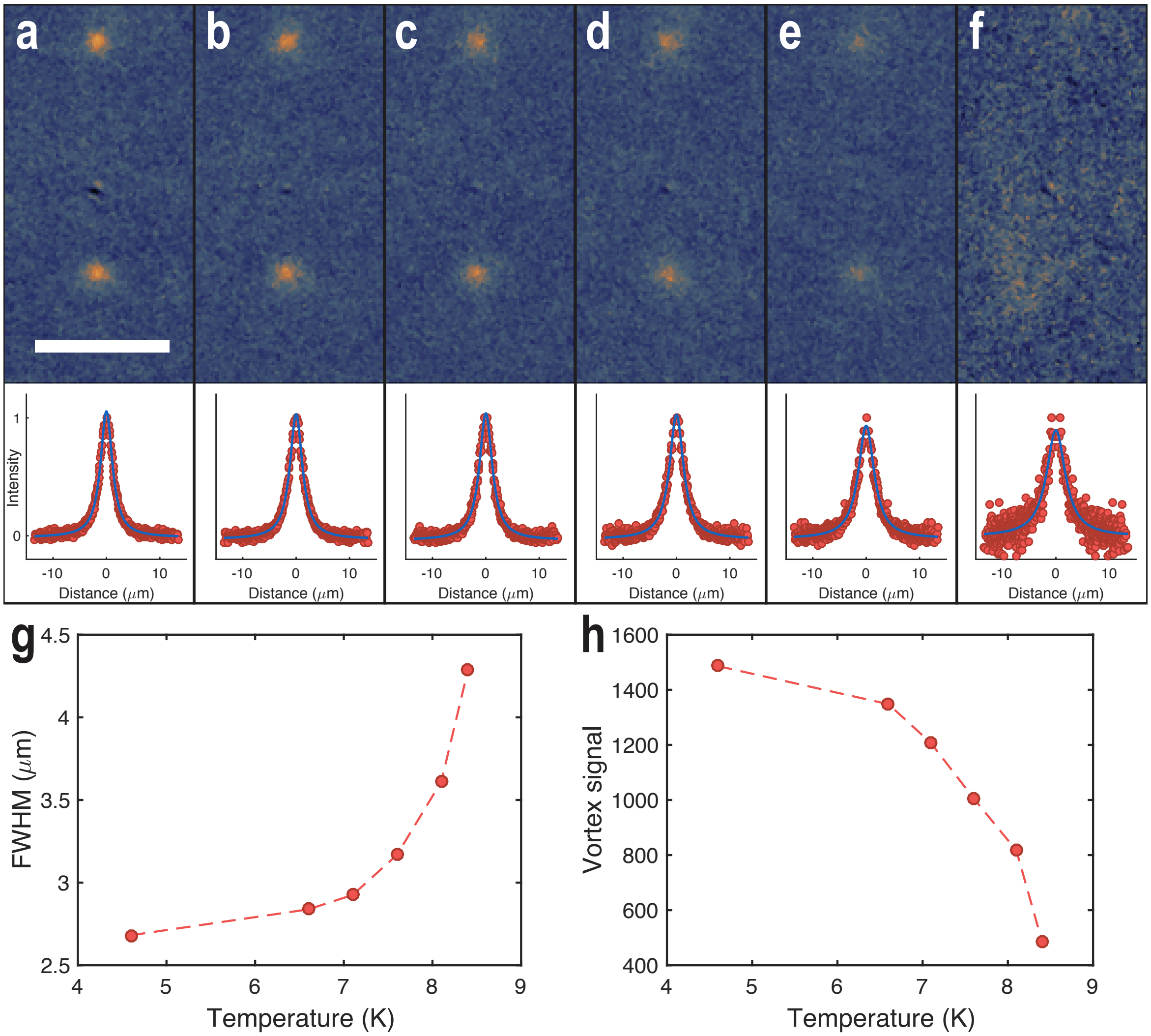

Supplementary Figure 1 : Vortex images at different SC temperatures.

Magneto-optical images of two vortices in our Niobium film at different SC temperatures ranging from 4.1 K to 8.4 K. The vortices were created under an external magnetic field H_{ext}=0.03 Oe. **a**, T=4.1 K. **b**, T=6.6 K. **c**, T=7.1 K. **d**, T=7.6 K. **e**, T=8.1 K. **f**, T=8.4 K. The scale bar is 20 µm. Below each frame is presented the corresponding normalized vortex intensity profile averaged over **a**, 11 vortices, **b**, 12 vortices, **c**, 12 vortices, **d**, 11 vortices, **e**, 12 vortices, **f**, 7 vortices. Each profile is fitted with a Lorentzian profile (solid curve). **g**, Temperature dependence of the full width at half maximum of the averaged vortex profile. **h,** Temperature dependence of the averaged vortex contrast. The optical resolution of vortex images at the lowest temperatures is ~ 2.5 µm, i.e. much larger than the diffraction limit (~0.6 µm). Indeed, it is set by the divergence of the magnetic field lines arising from the vortex since the Faraday rotation of light polarization is integrated in the whole indicator thickness. We see a slight increase in the vortex apparent size and a decrease in the magneto-optical contrast when the temperature is raised towards T_c. These evolutions are consistent with flux conservation expected in a vortex.

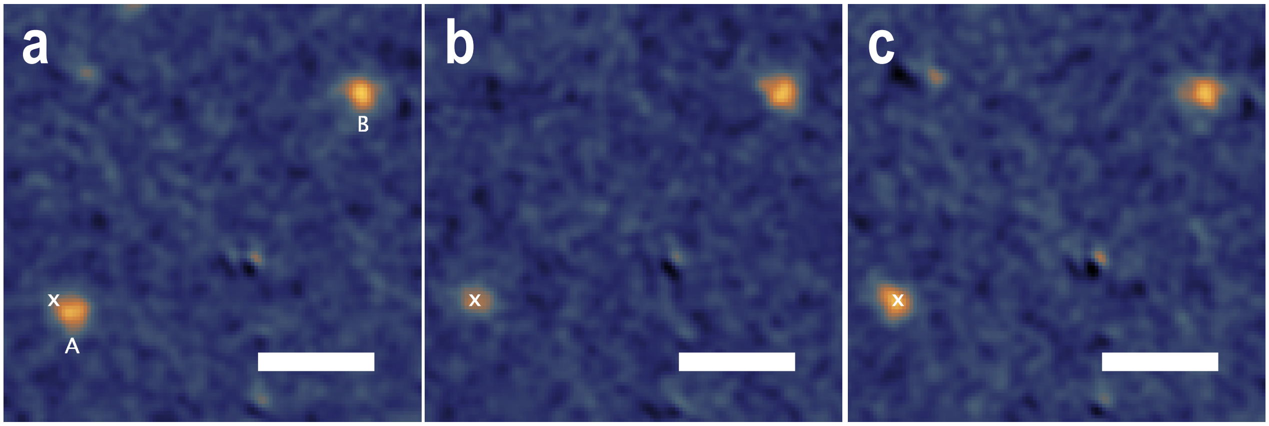

Supplementary Figure 2 : vortex survival under laser illumination.
a, Image of a vortex strongly bound to a pinning site (vortex A), at T=4.6 K. Vortex B is a reference vortex. **b**, Image of the same area under laser heating at the location marked with a white cross, with an absorbed power of 17 μW. **c**, Image of the same area after laser heating. The scale bar is 10 μm.

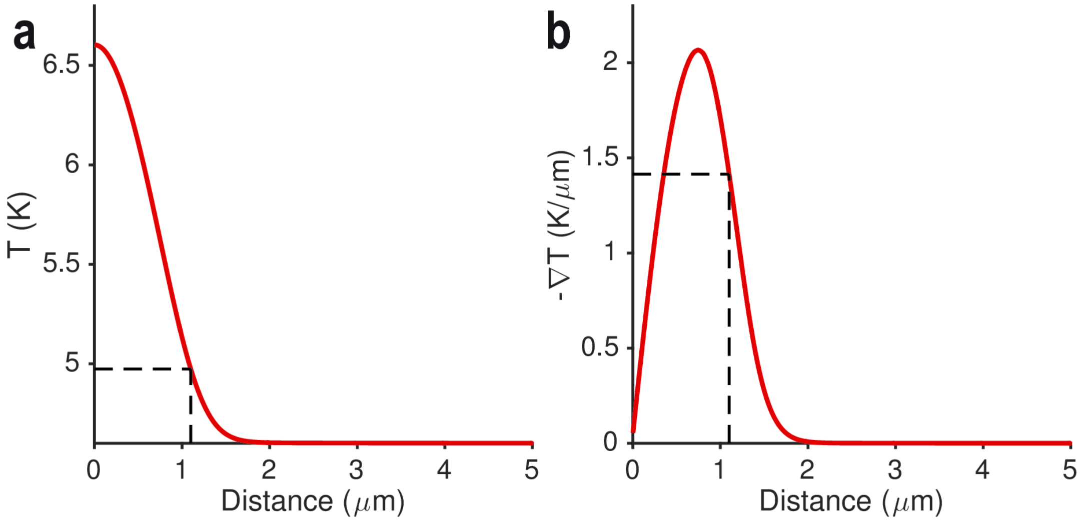

Supplementary Figure 3 : Temperature profile in the niobium film under laser heating.
For a vortex located at a distance r=1.1 μm from the center of the laser spot, the thermal gradient is estimated to 1.4 K.μm^{-1}, which leads to a thermal force per vortex unit length F = 14 pN/μm according to Eq. 2, taking ξ_0 = 10 nm and λ_0 = 90 nm.

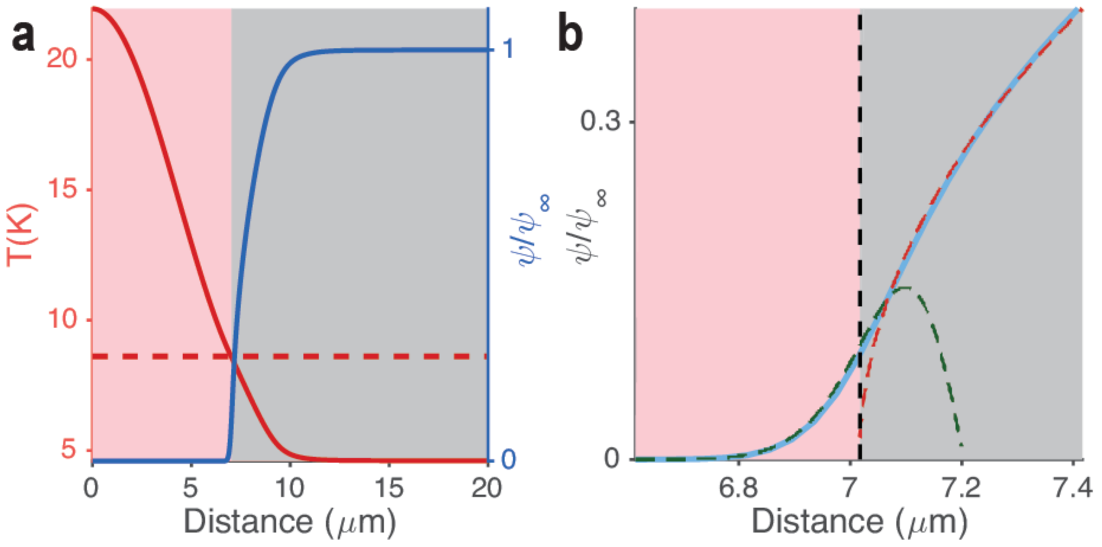

Supplementary Figure 4: Calculated order parameter profile across N/SC boundary
a, Red curve : calculated temperature profile just after the laser switch-off. The critical temperature of the niobium film (dashed level) is reached for a radius R_0 = 7 µm. Blue curve : numerical solution of the GL equation. **b**, Blue curve : Zoom of the numerical solution of the GL equation at the N/SC boundary. Dashed curves : Analytical solutions of the GL equation for $R = R_0$ at the foot of the order parameter front (green, Airy function comprising an exponential growth of Ψ) and in the SC region (red, square root evolution of Ψ).

Supplementary Note 1
Vortex manipulation with a laser which preserves superconductivity

We experimentally demonstrate that single vortices can be safely manipulated with a focused laser that does not destroy superconductivity. Supplementary Fig. 2a, b, c display the magneto-optical images of a strongly pinned vortex (named A) before, during and after illumination with a laser beam focused 1 μm away from the initial vortex position. The absorbed power is set to 17 μW, high enough to untrap this vortex and drop it at a new pinning site. The image of vortex A is slightly affected under illumination, meaning that the local temperature remains well below T_c when performing single vortex optical manipulation (see Supplementary Fig. 1).

Indeed, the absorption of visible photons by the superconductor certainly creates high-energy electrons that are harmful to Cooper pairs. This mechanism of pair-breaking is however limited to the very thin layer where light penetrates the superconductor[2] (the intensity skin depth, ~ 13 nm at visible wavelengths[3]). Since the thickness of this layer does not exceed the superconducting coherence length ξ_0 the superconductivity is not destroyed in films with a thickness much larger than ξ_0, which is the case here. This situation is similar to the proximity effect between a normal metal and a superconductor, where superconductivity is affected only in the close vicinity of the metal (at the ξ_0 scale).

Supplementary Note 2
Estimation of the temperature profile in the SC

The temperature profile created by a laser beam focused on our SC sample is calculated numerically using COMSOL program. This program solves the stationary heat equation taking into account temperature dependent thermal conductivities of the SC and the substrate (for instance 0.05 $W.m^{-1}.K^{-1}$ at T=4.6 K for niobium[1] and 450 $W.m^{-1}.K^{-1}$ at T=4.6 K for Si). We consider a Gaussian shaped heat source applied to the top of the niobium film:

$$Q(r) = \frac{P}{2\pi r_0^2} \exp\left[\frac{-r^2}{2r_0^2}\right]$$

where P is the total power absorbed by the SC and r_0 the size of the laser spot.

The Supplementary Fig. 3 shows the profiles of the temperature and the temperature gradient calculated for P= 13 μW, r_0=0.5 μm and a base temperature T = 4.6 K, matching the experimental conditions of Fig. 2. Clearly the whole temperature profile is below T_c.

Supplementary Note 3
Order parameter profile across the N/SC boundary

To study the evolution of vortex distribution after heating the SC above T_c with strong illumination we solve the Ginzburg-Landau (GL) equation to derive the spatial distribution of the order parameter for each temperature profile during the thermal relaxation.
Since the relaxation time of the order parameter ψ is much smaller than that of the temperature profile, we consider an adiabatic evolution of ψ, i.e $\partial_t \psi = 0$. Moreover, considering that the scale of the order parameter variation is much smaller than the hot-spot characteristic size, the term with the first derivative $\partial_r \psi$ can be neglected in $\Delta\ \psi$, so that the GL equation writes:

$$-\frac{\hbar^2}{4m}\frac{\partial^2\psi}{\partial r^2} + \alpha[T(r) - T_c]\psi + b\psi^3 = 0\ .$$

Far away from the laser induced hot spot, the temperature of the SC is equal to $T_0 < T_c$ and the order parameter ψ_∞ is uniform, determined by $\psi_\infty^2 = \alpha(T_c - T_0)/b$. Substituting the dimensionless order parameter $\Psi = \psi/\psi_\infty$ into the GL equation, we get:

$$-\frac{\hbar^2}{4m}\frac{\partial^2\Psi}{\partial r^2} + \alpha[T(r) - T_c]\Psi + \alpha(T_c - T_0)\Psi^3 = 0$$

After division by $\alpha(T_c - T_0)$ and introduction of the superconducting coherence length ξ_0 defined by $\xi_0^2 = \frac{\hbar^2}{4m\alpha T_c}$, the equation writes

$$-\frac{\xi_0^2}{1-T_0/T_c}\frac{\partial^2\Psi}{\partial r^2} + \frac{T(r)-T_c}{T_c-T_0}\Psi + \Psi^3 = 0,$$

with the boundary conditions $\psi(r=0) = 0$ and $\psi(r \longrightarrow \infty) = 1$.
The dimensionless form of this equation

$$\frac{\partial^2\Psi}{\partial\rho^2} = \tau(\rho)\Psi + \Psi^3, \text{ with } \rho = \frac{r}{\xi_0}\sqrt{1 - \frac{T_0}{T_c}} \text{ and } \tau(\rho) = \frac{T(\rho)-T_c}{T_c-T_0},$$

was numerically solved. The profile of the order parameter just after the laser switch-off is displayed in Supplementary Fig. 4a (blue curve).

Analytical solutions can be derived close to the circular N/SC moving boundary which is at a radius R. In this region the temperature is close to T_c and can be expressed using the expansion:

$$T(r) \approx T_c\left[1 - \frac{|\nabla T|_{r=R}}{T_c}(r - R)\right]$$

Defining the thermal length $L_T = T_c/|\nabla T|_{r=R}$, the GL equation takes the form:

$$-\xi_0^2\frac{\partial^2\Psi}{\partial r^2} - \frac{(r-R)}{L_T}\Psi + \left(1 - \frac{T_0}{T_c}\right)\Psi^3 = 0$$

i) In the region where $r - R \lesssim \xi_0$, the non-linear term can be neglected ($\Psi^3 \ll \Psi$) and the equation can be approximated by

$$\frac{\partial^2\Psi}{\partial r^2} + \frac{r-R}{L_T\xi_0^2}\Psi = 0$$

The order parameter is therefore proportional to the Airy function $Ai\left[-\frac{r-R}{L_\Psi}\right]$, where $L_\Psi = (\xi_0^2 L_T)^{\frac{1}{3}}$ is a characteristic thickness of the order parameter front.

ii) In the region where $r - R > \xi_0$, the typical scale of the order parameter variation is much larger than ξ_0 and the term $\xi_0^2 \partial_r^2 \Psi$ in the GL equation can be neglected. The order parameter thus evolves as $\sqrt{(r-R)/L_T}$. As shown in Supplementary Fig. 4b, the numerical profile of the order parameter at the boundary region can be well approximated with the analytical functions derived above.

Supplementary Note 4
Modelization of the magnetic field profile in the vortex cluster

Following the Bean critical state model, the vortex distribution is determined by the balance between the pinning force and the Lorentz driving force $j_c\Phi_0$, which pushes the vortices toward the SC region[6]. The radial profile of the vertical component of magnetic field, built from $j_c = -\frac{\partial H_z}{\partial r}$, is therefore linear in a thick superconductor. In order to match our experimental conditions, we consider here a SC film with finite thickness d, which occupies the region $-d < z < 0$. The current distribution in the vortex cluster with radius R^* can be modeled as

$$j_\theta = \begin{cases} j_c & \text{for} \quad r < R^* \\ 0 & \text{for} \quad r > R^* \end{cases} .$$

The vertical component of magnetic field created by this distribution in the plane z and at a radial distance r is given by:

$$H_z(r,z) = \frac{j_c}{2\pi}\int_z^{z+d} dz_0 \int_0^{R^*} \frac{1}{\sqrt{(a+r)^2+z_0^2}}\left[K\left(\frac{4ar}{(a+r)^2+z_0^2}\right) + \frac{a^2-r^2-z_0^2}{(a-r)^2+z_0^2}E\left(\frac{4ar}{(a+r)^2+z_0^2}\right)\right] da \ ,$$

where K and E are full elliptic integrals of the first and second kind, respectively[7].

In order to match the experimental observations, we calculate H_z at a height $z = 2.5$ µm from the niobium film and add to H_z a contribution which takes into account the averaged magnetic field H_v arising from the vortices imaged at $r > R_0$. This field is modeled with

$$H_\mathrm{v} = \frac{H_\mathrm{av}}{2}\left(1 + \mathrm{erf}\left(\frac{r-R_0}{\delta}\right)\right)$$

where H_av is a field amplitude and δ is the width of the magnetic field step, corresponding to our magneto-optical resolution. The experimental magnetic field profile is well reproduced by summing H_z and H_v (see Fig. 3d), taking $\delta = 2$ µm, R_0=7 µm, H_av=0.9 Oe.

Supplementary Note 5
Estimation of the geometrical barrier

The presence of the order parameter front provides a natural explanation for the vortex-free region surrounding the dense vortex cluster. After switching off the laser, the temperature starts to decrease and the N region shrinks. However, the magnetic flux stays trapped inside the N region until the magnetic field reaches the geometrical barrier critical value ($R = R^*$). The main physical idea of the geometrical barrier, introduced in Ref. [4] may be also applied to our case. Since the superconducting film thickness d is smaller than the London penetration depth λ, the magnetic field spreads from the vortex core over a distance $\lambda_{\mathrm{eff}} = \lambda^2/d$ larger than λ (see Ref. [5]). In this situation, the characteristic distance ζ from the N/SC interface at which the main variations of the vortex energy occur will be λ_{eff}. In our case, λ strongly varies with the distance from the N/SC boundary because of the temperature profile. Thus, the characteristic distance ζ should be estimated self-consistently by writing $\lambda_{\mathrm{eff}}(T) \sim \zeta$. Taking $\lambda \simeq \lambda_0\sqrt{L_{\mathrm{T}}/\zeta}$, we obtain $\zeta \sim \lambda_0\sqrt{(L_{\mathrm{T}}/d)}$.

As described in the Ref. [4], vortex penetration occurs when the Lorentz force $J\phi_0/c$ induced by the supercurrent density J reaches the value of $2\varepsilon_0/\zeta$, where $2\varepsilon_0 = \frac{\phi_0}{2\pi}\frac{H_{\mathrm{c1}}^0\zeta}{L_T}$ is the vortex line energy per unit length and H_{c1}^0 is the zero temperature critical field. This leads to :

$$\frac{J\phi_0}{c} = \frac{2\phi_0 H_{\mathrm{c1}}^0}{4\pi L_T} \quad .$$

By adapting the expression of the Meissner current J of Ref. [4] to our ring-shaped strip of width $R_0 - R^*$, which is valid in the limit $R_0 - R^* \ll R_0$, we obtain close to the N/SC boundary:

$$J(R^* + \zeta) \simeq \frac{cH_{\mathrm{a}}}{4\pi d}\sqrt{\frac{R_0 - R^*}{\zeta}} \quad .$$

In our configuration, the applied field H_{a} introduced in Ref. [4] corresponds to the field in the normal region which is given by $\left(\frac{R_0}{R^*}\right)^2 H_{\mathrm{ext}}$ due to flux conservation (field constriction). This leads to the following expression:

$$H_{\mathrm{ext}} \simeq 2H_{\mathrm{c1}}^0\left(\frac{R^*}{R_0}\right)^2\sqrt{\frac{\zeta}{(R_0 - R^*)}\frac{d}{L_T}} = 2H_{\mathrm{c1}}^0\left(\frac{R^*}{R_0}\right)^2\sqrt{\frac{d}{(R_0 - R^*)}}\left(\frac{{\lambda_0}^2 d}{{L_T}^3}\right)^{\frac{1}{4}} \quad .$$

The resolution of the heat equation leads to the temperature profile in our experimental conditions and yields $L_T \sim 5.3\ \mu m$. Taking $R_0 \sim 6.5$ µm and $R^* \sim 5.5\ \mu m$ (Fig. 3b-d), we obtain a penetration field of $6\ Oe$, which is in good agreement with $(R_0/R^*)^2 H_{\mathrm{ext}} = 4.3\ Oe$ from the experimental data. To adjust the experimental data of figure dependence of Fig. 3f, we thus used the formula

$$\frac{H_{\mathrm{ext}}}{H_{\mathrm{c1}}^0} = \frac{x^2}{\sqrt{1 - x}}\left(\frac{16{\lambda_0}^2 d^3}{{R_0}^2{L_T}^3}\right)^{\frac{1}{4}} \simeq 0.01\ \frac{x^2}{\sqrt{1 - x}} \quad .$$

Supplementary References

1. Koechlin, F. & Bonin, B. Parametrization of the niobium thermal conductivity in the superconducting state. *Supercond. Sci. Technol.* **9,** 453–460 (1996).
2. Testardi, L. R. Destruction of superconductivity by laser light. *Phys. Rev. B* **4,** 2189–2196 (1971).
3. Golovashkin, A. I., Leksina, I. E., Motulevich, G. P. & Shubin, A. A. Optical Properties of Niobium. *Sov Phys JETP* **29,** 27–34 (1969).
4. Zeldov, E., Larkin, A. I., Geshkenbein, V. B. & Konczykowski, M. Geometrical barriers in high-temperature superconductors. *Physical Review* **73,** 1428 (1994).
5. De Gennes, P. G. *Superconductivity of Metals and Alloys*. (Westview Press, 1999).
6. Tinkham, M. Introduction to superconductivity. McGraw–Hill (1996).
7. Jackson, J. D. *Classical Electrodynamics*. (Wiley, 1998).
